# Changes in sleep characteristics in Parkinson’s disease: a prospective 5-year follow-up study

**DOI:** 10.1007/s44470-026-00169-6

**Published:** 2026-09-11

**Authors:** Eemil Partinen, Ari Ylikoski, Christer Hublin, Markku Partinen

**Affiliations:** 1 Sleep Disorder Outpatient Clinic, Department of Psychiatry, Helsinki University Hospital, Helsinki, Finland; 2 Department of Clinical Neurosciences, Clinicum, University of Helsinki, Helsinki, Finland; 3 Helsinki Sleep Clinic, Terveystalo Healthcare, Helsinki, Finland; 4 The Social Insurance Institution of Finland (KELA), Helsinki, Finland; 5 Finnish Institute of Occupational Health, Helsinki, Finland

**Keywords:** Parkinson’s disease, Sleep disorders, Insomnia, Excessive daytime sleepiness, Follow-up, Sleep talking

## Abstract

**Purpose:**

Sleep disturbances are common and disabling symptoms in Parkinson’s disease (PD). Evolution of sleep disturbances in PD remains incompletely characterized. We aimed to describe changes in sleep parameters and sleep disorders over 5 years in a prospective PD cohort.

**Methods:**

A structured sleep questionnaire was administered to members of the Finnish Parkinson’s Association at baseline and at 5-year follow-up. Longitudinal changes were analyzed in 183 participants with complete data at both time.

**Results:**

Daytime sleepiness increased significantly, with the proportion of participants with Epworth Sleepiness Scale (ESS) > 10 rising from 30.6% to 38.8% (*P* = 0.026) and daily napping from 21.4% to 31.7% (*P* = 0.004). Sleep talking (≥ 1/week) increased from 17.4% to 30.4% (*P* < 0.001), nightmares from 14.8% to 24.6% (*P* = 0.003), and mean, REM Sleep Behavior Disorder Screening Questionnaire score (RBDSQ) from 4.4 to 5.3 points (*P* < 0.001). Restless legs syndrome (RLS) prevalence increased from 21.0% to 31.2% (*P* = 0.038). In contrast, insomnia symptoms, circadian timing and total sleep time remained stable. In multivariate logistic regression, excessive daytime sleepiness was independently associated with depressive symptoms, use of dopamine agonists, and risk of sleep apnea.

**Conclusion:**

Sleep disturbances in PD show a selective pattern of progression over 5 years. Daytime sleepiness, napping, RLS symptoms, sleep talking, nightmares and RBDSQ score increase while insomnia symptoms remain stable. These findings highlight the need for longitudinal, multi-domain sleep assessment in PD clinical care. The longitudinal increase in RBD-related symptoms and sleep talking strengthens their utility as markers of disease progression and clinical prognostic relevance.

**Brief summary:**

**Current knowledge/study rationale:**

Sleep disturbances are among the most frequent and disabling non-motor symptoms of Parkinson’s disease. These include insomnia, excessive daytime sleepiness, REM sleep behavior disorder, and restless legs syndrome. Long-term prospective data on how multiple sleep disorders and sleep characteristics evolve over time in PD is lacking.

**Study impact:**

In this 5-year prospective cohort study sleep disturbances in PD progress selectively. Daytime sleepiness, napping, restless legs syndrome, sleep talking, nightmares and RBD-related features worsen significantly while insomnia symptoms remain stable. This provides evidence that sleep characteristics and disorders evolve in time. Sleep disturbances in PD should be assessed routinely in clinical practice.

**Graphical Abstract:**

**Figure:**
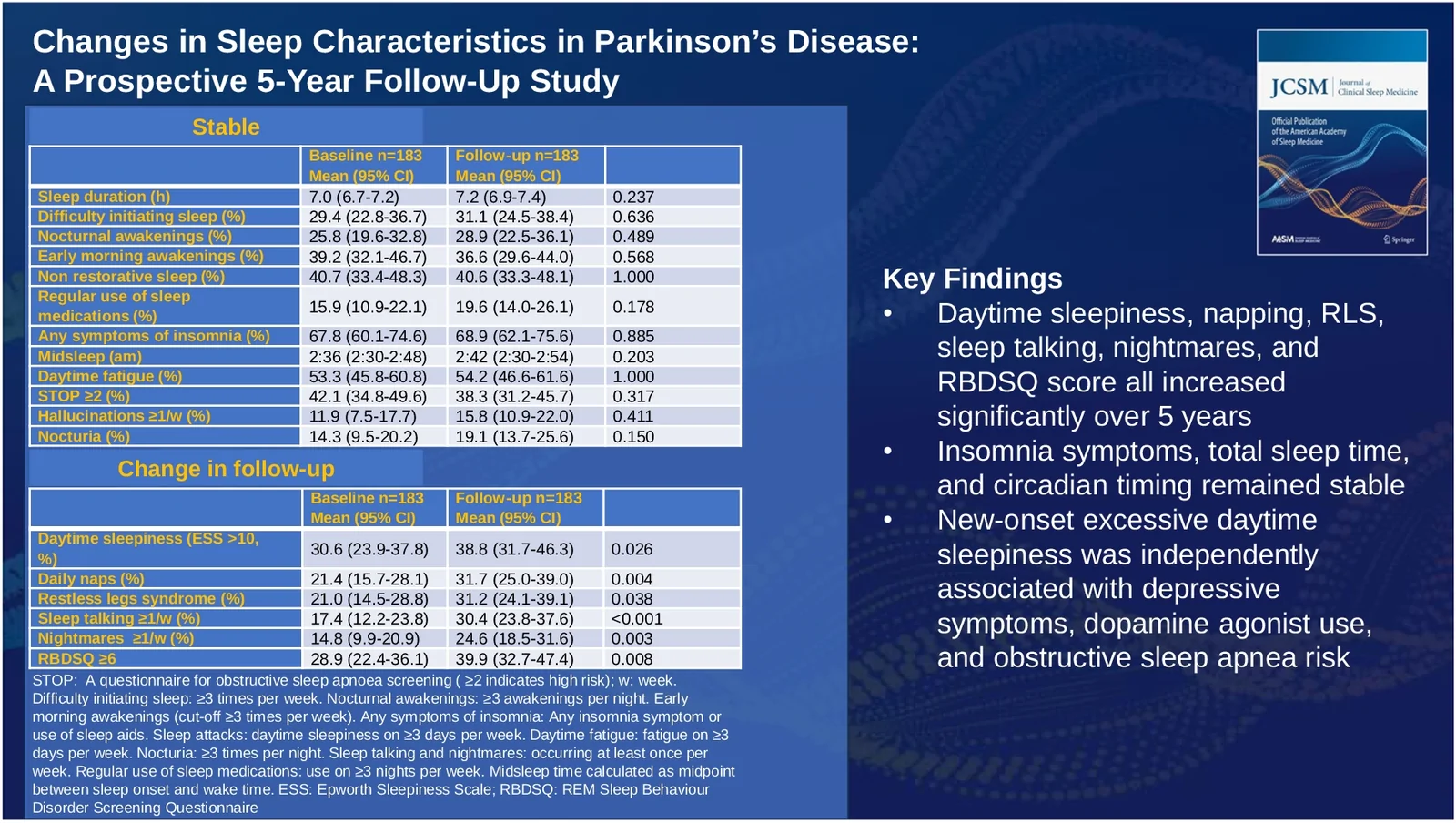

**Supplementary Information:**

The online version contains supplementary material available at https://doi.org/10.1007/s44470-026-00169-6.

## Introduction

Sleep disturbances are among the most frequent non-motor symptoms of Parkinson’s disease (PD) [1, 2]. They may emerge years before motor symptom onset and tend to worsen as the disease progresses [3–5]. A wide range of sleep-related problems are observed in PD, including insomnia, excessive daytime sleepiness, REM sleep behavior disorder (RBD) and restless legs syndrome (RLS) [2, 6, 7]. These disturbances can interact with each other and with other non-motor symptoms such as depression, fatigue, and cognitive decline, substantially affecting quality of life [2, 8, 9].

Shortening of sleep duration, deterioration of sleep and longer sleep duration have been associated with an increased risk of developing PD [4, 10]. In our previous work within the same cohort, we demonstrated that sleep talking, a potential marker of REM sleep behavior disorder (RBD), and longer sleep duration were both linked to higher mortality in patients with PD [7, 11]. These findings suggest that alterations in sleep duration and specific sleep-related behaviors may reflect underlying neurodegenerative processes relevant to disease prognosis.

However, most earlier studies have examined single sleep symptoms in isolation, typically in cross-sectional or short-term designs. As a result, the long-term evolution and interrelationships of multiple sleep disorders and sleep parameters in PD remain incompletely understood.

To address this gap, we conducted a 5-year prospective follow-up study to characterize changes in key sleep parameters and disorders, including sleep duration, chronotype, insomnia, RLS, snoring, sleep talking, RBD and sleep apnea.

We hypothesized that sleep duration and daytime sleepiness would increase during 5 years, and that the prevalence of sleep talking would rise in parallel with disease progression.

## Methods

From the 7500 members of the Finnish Parkinson’s Association 5373 were registered as PD patients. A random sample of 1500 subjects was selected from these registered PD patients. A structured questionnaire was sent to 1447 eligible participants between 2010 and 2011. Inclusion criteria were Parkinson’s disease diagnosis confirmed by a neurologist, ability to consent, and being alive at baseline. In Finland, PD diagnosis is established by a specialist neurologist and follows national clinical guidelines consistent with the Gelb diagnostic criteria [12, 13]. Diagnoses were self-reported as neurologist-confirmed and were not re-verified by medical record review. The questionnaire was designed for PD patients, with sleep-related items from the validated Basic Nordic Sleep Questionnaire (BNSQ) [14]. Further details of the cohort and survey have been published previously [15]. Consenting participants were sent a follow-up questionnaire in 2014 to 2015. Non-respondents were reminded and telephoned. Altogether, 183 subjects responded to all questions used in the study.

Age was calculated from date of birth to questionnaire date. Gender was recorded as female or male. Body mass index (BMI) was calculated from self-reported height and weight. Health-related quality of life was defined as poor when the standardized WHO-5 Well-Being Index score was < 50 [16]. Education level was determined based on total duration of school education (basic and higher). Weekly metabolic equivalent values in minutes (MET-minutes) were derived from self-reported physical activity. Participants were categorized as sedentary (< 100), moderately active (100 – < 1000), or highly active (≥ 1000 MET-minutes) [17].

Total daily levodopa equivalent dose (LED) was computed using standard conversion factors for each self-reported medication [18]. Olfactory dysfunction was defined as a subjectively reduced sense of smell compared to others, constipation as infrequent bowel movements (≤ 3 per week), and nocturia as the need to urinate ≥ 3 times per night. Depressive symptoms were screened with Rimon’s Brief Depression Scale, using a score ≥ 10 to indicate depression. The scale excludes insomnia symptoms, is validated against the Beck Depression Inventory, and is available in Finnish [19]. Regular use of sleep medication was defined as use on ≥ 3 nights per week. Sleep attacks were measured with a question “Have you suffered from a compulsive tendency to fall asleep (an irresistible urge to fall asleep) on weekdays/working days?”. Sleep attacks was considered present if they were reported on ≥ 3 days per week. We also used the Epworth Sleepiness Scale (ESS) to assess sleepiness [20]. Daytime fatigue was assessed using a question “Have you felt tired during the daytime?”. Cut-off for fatigue was ≥ 3 days per week. Sleep-disordered breathing risk was assessed with the STOP questionnaire, using a score ≥ 2 as the cutoff for high risk of sleep apnea [21]. Insomnia symptoms such as difficulty initiating sleep (cut off ≥ 3 times per week), nocturnal awakenings during the night (cut off ≥ 3 times per night), early morning awakenings (cut off ≥ 3 times per week) and non-restorative sleep were assessed on a five-point frequency scale (as in BNSQ). A binary composite variable capturing any insomnia symptom was defined as the presence of at least one of the following: Difficulty initiating sleep, nocturnal awakenings, early morning awakenings, non-restorative sleep, or regular use of sleep aids. RLS was defined according to the international 4-item definition criteria [22]. REM sleep behavior disorder (RBD) symptoms were screened with the RBD Screening Questionnaire (RBDSQ), with a score ≥ 6 indicating RBD [23]. We used insomnia severity index (ISI) to assess insomnia symptoms at follow-up [24]. Unfortunately, we did not include ISI to baseline questionnaire.

Total sleep time (TST) was calculated from self-reported median sleep duration per 24 h, excluding naps. Circadian timing was estimated using midsleep, a midpoint of sleep calculated from bedtime, sleep latency and time to wake up.

Statistical analyses were performed using STATA 19.5 (StataCorp, USA). Descriptive statistics for continuous variables are presented as means with 95% confidence intervals (CI). The Shapiro–Wilk test was conducted to check for normality in the distributions. To assess potential selection bias, we compared baseline characteristics of follow-up cohort (*n* = 183) with the full baseline cohort (*n* = 611). Independent-samples comparisons were performed using the Mann–Whitney two sample statistic test for continuous variables and the chi-square test for categorical variables, with Fisher’s exact test applied where expected cell counts were less than five. Longitudinal changes in continuous and categorical variables were compared using the Wilcoxon signed-rank test. To identify clinical and demographic factors associated with excessive daytime sleepiness at follow-up, we performed univariate and multivariate logistic regression analyses with ESS > 10 as the dependent variable. Daytime sleepiness was selected as the primary outcome for regression analysis because it showed a clinically and statistically significant increase over the follow-up period and is a symptom of particular functional relevance in PD. All candidate variables were measured at follow-up and included age, sleep duration, depression score, LED, use of dopamine agonists, risk of sleep apnea, RBDSQ-score. All variables were entered into the multivariate model. Results are presented as odds ratios (OR) with 95% CI. Statistical significance was set at *P* < 0.05.

## Results

Subjects in the follow-up cohort were younger, had shorter disease duration, higher BMI, and better health-related quality of life compared to those who did not complete follow-up or had missing data. Gender, LED, olfactory dysfunction, constipation, and depression prevalence did not differ significantly between groups. Please see Supplementary Table 1 for a comparison of baseline characteristics between included and excluded subjects.

Demographics and other characteristics of the cohort are presented in Table 1.

**Table 1:** Characteristics of the cohort

	All at baseline; ***N*** = 611 (95% CI)	Baseline (5 y fup cohort); *n* = 183 (95% CI)	Follow-up (5 y fup cohort); *n* = 183 (95% CI)	*P*-value (Baseline vs fup)
Age (years)	67.9 (66.2–68.6)	65.6 (64.4–66.8)	69.7 (68.5–70.9)	< 0.001*
Gender (men, %)	54.2 (50.1–58.2)	52.5 (45.0–59.9)	52.5 (45.0–59.9)	1
BMI (kg/m^2^)	26.5 (26.1–26.8)	27.2 (26.5–27.9)	26.8 (26.0–27.5)	0.051
Education ≤ 9 years (%)	41.0 (37.0–45.0)	38.3 (31.2–45.7)	38.3 (31.2–45.7)	1
Education > 9 to ≤ 12 years (%)	42.1 (38.1–46.2)	45.4 (38.0–52.9)	45.4 (38.0–52.9)	1
Education > 12 years (%)	16.9 (14.0–20.1)	16.4 (11.3–22.6)	16.4 (11.3–22.6)	1
Mean physical activity (MET-min)	571.7 (524.2–619.1)	600.7 (506.9–694.5)	469.0 (401.6–536.5)	0.022*
Sedentary (MET < 100, %)	15.6 (12.8–18.8)	9.0 (5.3–14.3)	18.0 (12.8–24.4)	0.008*
Active (MET ≥ 100 & < 1000, %)	67.9 (64.0–71.7)	74.6 (67.5–80.1)	69.4 (62.2–76.0)	0.317
Highly active (MET ≥ 1000, %)	16.4 (13.6–19.7)	16.4 (11.3–22.7)	12.6 (8.1–18.3)	0.317
Duration of PD (years)	6.3 (5.9–6.6)	5.5 (5.0–6.0)	9.7 (9.1–10.2)	< 0.001*
LED (mg)	644.7 (612.4–677.0)	607.8 (558.0–657.6)	732.6 (675.2–790.1)	< 0.001*
Olfactory dysfunction (%)	67.3 (63.4–71.0)	62.8 (55.4–69.9)	71.0 (63.9–77.5)	0.005*
Constipation	31.6 (27.9–35.4)	29.0 (22.5–36.1)	31.7 (25.0–39.0)	0.484
Poor quality of life (WHO5 < 50, %)	42.2 (38.3–46.3)	33.9 (27.1–41.2)	38.8 (31.7–46.3)	0.225
Depression (%)	27.1 (23.6–30.8)	22.5 (16.7–29.3)	23.5 (17.6–30.3)	0.763

*PD* Parkinson’s disease, *BMI* body mass index, *LED* levodopa equivalent dose, *WHO-5* WHO-5 Well-Being Index, *MET* metabolic equivalent of task; BMI calculated from self-reported height and weight. Physical activity categories: sedentary (MET-minutes/week < 100), moderately active (100 to < 1000), highly active (≥ 1000). LED calculated using standard conversion factors. Olfactory dysfunction defined as subjectively reduced sense of smell compared to others. Constipation defined as ≤ 3 bowel movements per week. Poor quality of life defined as WHO-5 Well-Being Index score < 50. Depression screened using Rimon’s Brief Depression Scale (cut-off ≥ 10); the scale excludes insomnia items and is validated against the Beck Depression Inventory

^*^Statistical significance *P* < 0.05. *P*-value calculated using the Wilcoxon signed-rank test

Mean disease duration increased from 5.5 to 9.7 years (*P* < 0.001). Education levels remained unchanged during follow-up, as no participants pursued further studies. Physical activity decreased during follow-up, with the proportion of sedentary participants rising from 9.0% to 18.0% (*P* = 0.008). Mean levodopa equivalent dose (LED) increased from 607.8 mg to 732.6 mg (*P* < 0.001), and olfactory dysfunction increased from 62.8% to 71.0% (*P* = 0.005). Constipation, depression, and health-related quality of life (WHO-5) remained unchanged during follow-up.

Changes in sleep characteristics are shown in Table 2. Sleep duration showed a non-significant increase from 7.0 to 7.2 h (*P* = 0.237). Individual insomnia symptoms (difficulty initiating sleep, nocturnal awakenings, early morning awakenings, and non-restorative sleep) did not change significantly over the follow-up period. Also, the composite variable capturing any insomnia symptoms or use of sleep aids remained unchanged. Similarly, daytime fatigue and hallucinations remained stable. In contrast, daytime sleepiness increased significantly: the proportion of participants with an ESS score > 10 rose from 30.6% to 38.8% (*P* = 0.026), and severe sleepiness (ESS > 15) from 8.9% to 14.8% (*P* = 0.043). Daily napping increased from 21.4% to 31.7% (*P* = 0.004). Restless legs syndrome (RLS) prevalence increased from 21.0% to 31.2% (*P* = 0.038). Sleep talking (≥ 1/week) increased markedly from 17.4% to 30.4% (*P* < 0.001), and nightmares (≥ 1/week) from 14.8% to 24.6% (*P* = 0.003). Mean RBDSQ score increased from 4.4 to 5.3 points (*P* < 0.001), and the proportion meeting the RBDSQ ≥ 6 threshold rose from 28.9% to 39.9% (*P* = 0.008). Midsleep time and sleep-disordered breathing (OSA) risk (STOP ≥ 2) were unchanged.

**Table 2:** Change of sleep disorders and characteristics in 5-year follow-up

	Baseline, *n* = 183 Mean (95% CI)	Follow-up, *n* = 183 Mean (95% CI)	*P*-value
Sleep duration (h)	7.0 (6.7–7.2)	7.2 (6.9–7.4)	0.237
Difficulty initiating sleep (%)	29.4 (22.8–36.7)	31.1 (24.5–38.4)	0.636
Nocturnal awakenings (%)	25.8 (19.6–32.8)	28.9 (22.5–36.1)	0.489
Early morning awakenings (%)	39.2 (32.1–46.7)	36.6 (29.6–44.0)	0.568
Non restorative sleep (%)	40.7 (33.4–48.3)	40.6 (33.3–48.1)	1.000
Regular use of sleep medications (%)	15.9 (10.9–22.1)	19.6 (14.0–26.1)	0.178
Any symptoms of insomnia (%)	67.8 (60.1–74.6)	68.9 (62.1–75.6)	0.885
Midsleep (am)	2:36 (2:30–2:48)	2:42 (2:30–2:54)	0.203
Sleep attacks (%)	25.3 (19.1–32.3)	28.3 (21.9–35.5)	0.189
ESS > 10 (%)	30.6 (23.9–37.8)	38.8 (31.7–46.3)	0.026*
ESS > 15 (%)	8.9 (5.2–14.0)	14.8 (10.0–20.7)	0.043*
Daily naps (%)	21.4 (15.7–28.1)	31.7 (25.0–39.0)	0.004*
Daytime fatigue (%)	53.3 (45.8–60.8)	54.2 (46.6–61.6)	1.000
RLS (%)	21.0 (14.5–28.8)	31.2 (24.1–39.1)	0.038*
STOP ≥ 2 (%)	42.1 (34.8–49.6)	38.3 (31.2–45.7)	0.317
Sleep talking ≥ 1/w (%)	17.4 (12.2–23.8)	30.4 (23.8–37.6)	< 0.001*
Nightmares ≥ 1/w (%)	14.8 (9.9–20.9)	24.6 (18.5–31.6)	0.003*
Hallucinations ≥ 1/w (%)	11.9 (7.5–17.7)	15.8 (10.9–22.0)	0.411
Nocturia (%)	14.3 (9.5–20.2)	19.1 (13.7–25.6)	0.150
RBDSQ (points)	4.4 (4.0–4.8)	5.3 (4.8–5.7)	< 0.001*
RBDSQ ≥ 6	28.9 (22.4–36.1)	39.9 (32.7–47.4)	0.008*

*ESS* Epworth Sleepiness Scale, *RLS* Restless legs syndrome, *STOP* STOP questionnaire for obstructive sleep apnea screening (score ≥ 2 indicates high risk), *RBDSQ* REM Sleep Behavior Disorder Screening Questionnaire, *w* week

Difficulty initiating sleep: ≥ 3 times per week. Nocturnal awakenings: ≥ 3 awakenings per night. Early morning awakenings and non-restorative sleep assessed on a 5-point frequency scale (cut-off ≥ 3 times per week). Any symptoms of insomnia: Any insomnia symptom or use of sleep aids. Sleep attacks: daytime sleepiness on ≥ 3 days per week. Daytime fatigue: fatigue on ≥ 3 days per week. Nocturia: ≥ 3 times per night. Sleep talking and nightmares: occurring at least once per week. Regular use of sleep medications: use on ≥ 3 nights per week. Midsleep time calculated as midpoint between sleep onset and wake time

^*^Statistical significance *P* < 0.05. *P*-values calculated using the Wilcoxon signed-rank test

At follow-up, the mean ISI score was 8.7 (95% CI 7.8–9.5). Applying standard severity thresholds, the majority, 56.9% (95% CI 49.3–64.5), had no clinically significant insomnia (ISI < 10), 28.1% (95% CI 21.3–35.0) had subthreshold insomnia (ISI 10–14), and 15.0% (95% CI 9.5–20.4) met criteria for clinical insomnia (ISI ≥ 15).

Results for logistic regression models for excessive sleepiness at follow up (ESS > 10) are presented in Table 3. In the fully adjusted model depression score, use of dopamine agonist and risk of sleep apnea were associated with excessive daytime sleepiness.

**Table 3:** Logistic regression analysis for excessive daytime sleepiness (ESS > 10)

	Univariate model, *n* = 183 Odds ratio (95% CI)	*P*-value	Multivariate model, *n* = 183 Odds ratio (95% CI)	*P*-value
Age	0.98 (0.94–1.01)	0.185	0.97 (0.93–1.01)	0.184
Male gender	1.12 (0.61–2.07)	0.716	1.22 (0.60–2.46)	0.587
Sleep duration	0.97 (0.83–1.14)	0.720	0.99 (0.81–1.20)	0.894
RBDSQ score	1.13 (1.03–1.25)	0.011*	1.04 (0.93–1.17)	0.490
Depression score	1.15 (1.06–1.24)	< 0.001*	1.18 (1.07–1.30)	0.001*
LED	1.00 (1.00–1.00)	0.689	1.00 (1.00–1.00)	0.925
Use of dopamine agonist	1.98 (1.01–5.21)	0.047*	3.94 (1.54–10.04)	0.004*
STOP	1.71 (1.25–2.33)	0.001*	1.54 (1.08–2.20)	0.017*

*RBDSQ* REM Sleep Behavior Disorder Screening Questionnaire; Depression score: Rimon’s Brief Depression Scale as continuous variable, the scale excludes insomnia items and is validated against the Beck Depression Inventory; *LED* levodopa equivalent dose, *STOP* STOP questionnaire for obstructive sleep apnea screening

^*^Statistical significance *P* < 0.05

In sensitivity analyses, the association between dopaminergic medication use and excessive daytime sleepiness at follow-up was examined in univariate logistic regression models. Neither levodopa OR 0.55 (95% CI 0.18–1.70, *P* = 0.295) nor MAO inhibitors OR 0.98 (95% CI 0.52–1.83, *P* = 0.945) were associated with excessive daytime sleepiness. When use of different dopaminergic medication was added to the fully adjusted logistic regression model, higher depression score (OR 1.18, 95% CI 1.07–1.31, *P* = 0.001), high risk of sleep apnea (STOP ≥ 2; OR 1.53, 95% CI 1.05–2.21, *P* = 0.025), and use of dopamine agonists (OR 3.92, 95% CI 1.41–10.86, *P* = 0.009) were independently associated with excessive daytime sleepiness. Age (OR 0.97, 95% CI 0.92–1.01, *P* = 0.165), total sleep time (OR 0.95, 95% CI 0.77–1.16, *P* = 0.595), RBDSQ score (OR 1.08, 95% CI 0.95–1.22, *P* = 0.247), MAO inhibitor use (OR 0.82, 95% CI 0.38–1.77, *P* = 0.611) and levodopa use (OR 0.52, 95% CI 0.15–1.87, *P* = 0.319) were not significantly associated with excessive daytime sleepiness.

## Discussion

We observed changes in sleep characteristics over a 5-year follow-up period. The most relevant findings were an increase in daytime sleepiness, greater daily napping, a higher prevalence of RLS symptoms, and a marked rise in sleep talking, nightmares, and RBDSQ scores. In contrast, insomnia symptoms, total sleep time, midsleep as a marker of circadian timing and sleep-disordered breathing risk remained stable over the follow-up period. These findings support the idea that specific sleep disturbances in PD may change in time. Contrary to previous findings we did not observe an increase in symptoms of insomnia.

Total sleep time did not change significantly over 5 years, increasing from 7.0 to 7.2 h (*P* = 0.237). This is broadly consistent with the general pattern of age-related sleep changes. In general population also sleep efficiency declines and nocturnal awakenings become more frequent [25]. However, we did not observe a significant increase in nocturnal awakenings or early morning awakenings, which may partly reflect a survivor effect in our follow-up cohort. The absence of a significant changes in sleep duration is noteworthy given that both prolonged and shorter sleep has been associated with cognitive decline and increased dementia risk in prior studies and longer sleep duration has been linked to higher mortality in this cohort [11, 26].

A contrast to previous finding is that individual insomnia symptoms (difficulty initiating sleep, nocturnal awakenings, early morning awakenings, and non-restorative sleep) did not increase during follow-up. This contrasts with some earlier reports describing insomnia as a common and progressive complaint in PD [5]. A composite variable capturing any insomnia symptom or regular use of sleep aids also remained stable over the follow-up period, suggesting that the modest increase in sleep aid use (15.9% at baseline vs 19.6% at follow-up) does not explain the stability of insomnia symptoms. A possible explanation is that, as disease severity increases, patients may become less distressed by or less aware of fragmented sleep. The availability of ISI scores only at follow-up limits direct longitudinal comparison of insomnia severity using a validated instrument, which should be addressed in future studies. Circadian timing was stable over follow-up period, but circadian propensity is not easily discernible from insomnia features in our questionnaire data.

The progression of RBD-related features, sleep talking and nightmares is among the most clinically significant findings of this study. Sleep talking (≥ 1/week) nearly doubled in prevalence from 17.4% to 30.4% (*P* < 0.001), and nightmares increased from 14.8% to 24.6% (*P* = 0.003). Mean RBDSQ score increased significantly, and the proportion meeting the RBDSQ ≥ 6 threshold rose from 28.9% to 39.9% (*P* = 0.008). RBD is recognized as a core prodromal feature of synucleinopathies and is strongly associated with subsequent cognitive decline and a more malignant PD phenotype [3]. The increase in RBDSQ scores suggests active progression of REM sleep pathology as PD advances. We have previously reported that sleep talking is associated with increased mortality in this cohort [7]. The present longitudinal data reinforce its clinical significance as a marker of disease progression rather than an incidental finding.

Daytime sleepiness increased significantly over 5 years, with a clinically meaningful rise in both pooled excessive sleepiness (ESS > 10) and severe (ESS > 15) sleepiness. The prevalence is in line with other studies [27]. Similarly, daily napping increased, possibly reflecting higher daytime sleepiness. Sleepiness was associated with symptoms of depression, use of dopaminergic drugs and risk of sleep apnea. Other factors may also have contributed to the observed increase in sleepiness, including changes in medications not captured in our regression model, comorbidities, and changes in societal well-being and daily functioning. Nevertheless, this finding highlights the need for addressing secondary reasons for sleepiness.

RLS prevalence increased significantly from 21.0% to 31.2% over 5 years (*P* = 0.038). RLS in PD shares dopaminergic pathophysiology, although RLS has several other proposed symptom generating mechanisms as well. For example, in GWAS, studies mainly non-dopaminergic risk loci have been identified [28]. Progression of RLS symptoms alongside increasing LED could reflect inadequate drug treatment but also augmentation. The increase in RLS prevalence is broadly consistent with cross-sectional reports of elevated RLS frequency in advanced PD [29, 30].

The stability of sleep-disordered breathing risk (STOP ≥ 2) over 5 years is somewhat unexpected given the progressive motor and autonomic dysfunction in PD. One plausible explanation is that weight loss (reflected by the trend towards lower BMI at follow-up *P* = 0.051) changes the STOP-score. We highlight that STOP questionnaire captures self-reported symptoms and may lack sensitivity for detecting changes in OSA severity in this population. Objective sleep polygraphy data would be needed to confirm this finding.

## Strengths and limitations

The prospective design with a 5-year interval is a key strength, enabling genuine longitudinal characterization of sleep disturbances in an established PD cohort. The use of a structured, validated sleep questionnaire (BNSQ) applied consistently at both time points enhances comparability. The inclusion of multiple sleep domains (insomnia, sleepiness, parasomnias, RLS, chronotype, and sleep-disordered breathing) provides a comprehensive picture of the possible sleep phenotype in PD [31].

Several limitations merit consideration. The follow-up sample (*n* = 183) is small relative to the baseline cohort (*n* = 611), as only participants with complete data at both time points were included. This choice introduces a survivor effect: patients who died or were too unwell to complete the follow-up questionnaire are systematically excluded. This bias is supported by finding that subjects in the follow-up cohort were younger, had better QOL and shorter PD duration. This healthy-survivor bias could attenuate the true magnitude of changes in sleep duration, sleepiness, and parasomnias over time, meaning our estimates are probably conservative. We did not have motor symptom measures available, which prevents us from correlating changes in sleep characteristics with changes in core motor symptoms. The baseline sample was randomly selected from the Finnish Parkinson’s Association registry, which covers a large portion of PD patients in Finland, and is considered broadly representative of the Finnish PD population. However, this may result in selection bias towards more active or less severely affected patients. The absence of a healthy control population limits our ability to dissect disease-specific effects from normal age-related sleep changes. As the study was conducted in Finland, cultural differences and healthcare practices may limit the generalizability of these findings to other populations and healthcare settings.

Recall bias may affect self-reported sleep parameters, although its direction is likely consistent across both time points. The reliance on a questionnaire-based approach, without objective sleep recording, means that some diagnoses, particularly RBD and sleep apnea are based on surrogate markers. Also, sleep talking and nightmares could appear as isolated symptoms without relation to RBD. Video-polysomnography would be required for accurate classification of RBD, OSA and other parasomnias. Similarly, PD diagnoses were self-reported as established by a neurologist at baseline but not reverified at follow-up or by reviewing medical records. This is a limitation of large registry-based cohort studies. In Finland, PD diagnosis is largely given by a movement disorder expert or specializing physician under expert supervision. Diagnosis established by a specialist neurologist is a structural requirement of the healthcare system. The national diagnostic criteria applied during the enrolment period (2010–2011) were consistent with the Gelb criteria [12, 13]. Few participants may have received an alternative or revised diagnosis over time and forgot to inform us via the questionnaire. The ISI score was only included at follow-up, forbidding direct longitudinal comparison of insomnia severity using this validated tool.

## Conclusions

In this prospective cohort study of patients with Parkinson’s disease, sleep disturbances showed a selective pattern of progression over 5 years. Daytime sleepiness, daily napping, symptoms of RLS, sleep talking, nightmares, and RBDSQ scores increased significantly. In contrast, insomnia symptoms, circadian timing, total sleep duration, and sleep-disordered breathing risk remained unchanged. The increase in sleepiness was associated with symptoms of depression, use of dopamine agonist and the risk of sleep apnea, highlighting the need for addressing these factors in Parkinson patients suffering from sleepiness. The longitudinal increase in RBD-related symptoms and sleep talking strengthens their utility as markers of disease progression and clinical prognostic relevance.

## Supplementary Information

Below is the link to the electronic supplementary material.

ESM 1 (DOCX 16.2 KB)

## Data Availability

The data that support the findings of this study are available from the corresponding author upon reasonable request.
