# Supplementary material for: Changes in sleep characteristics in Parkinson’s disease: a prospective 5-year follow-up study
Source: J Clin Sleep Med. 2026 Sep 11;22(1):166. doi: 10.1007/s44470-026-00169-6 (PMC13569764; doi:10.1007/s44470-026-00169-6)
Supplement: Supplementary file 1 [file 44470_2026_169_MOESM1_ESM.docx]

### Supplement Table 1 Characteristics of included and excluded participants

|  | Included  (n=183) | Excluded  (number varies due to missing data n=) | P-value |
| --- | --- | --- | --- |
| Age (years) | 65.6 (64.4-66.8) | 69.9 (69.2-70.5) | <0.001* |
| Male gender (%) | 52.5 (45.0-59.9) | 52.9 (49.0-56.8) | 0.933 |
| BMI (kg/m^2^) | 27.2 (26.5-27.9) | 26.3 (25.0 – 26.6) | 0.015* |
| Duration of PD (years) | 5.5 (5.0-6.0) | 6.7 (6.3-7.0) | 0.015* |
| LED (mg) | 607.8 ( 558.0-657.6) | 636 (603-670) | 0.600 |
| Olfactory dysfunction (%) | 62.8 (55.4-69.9) | 68.1 (64.3-71.8) | 0.211 |
| Constipation (%) | 29.0 (22.5-36.1) | 32.7 (29.1-36.6) | 0.367 |
| Poor quality of life (WHO5 < 50, %) | 33.9 (27.1-41.2) | 46.1 (42.2-50.1) | 0.003* |
| Depression (%) | 22.5 (16.7-29.3) | 29.0 25.5-32.8) | 0.090 |

BMI: Body Mass Index; PD: Parkinson disease; LED: Levodopa equivalent dose; WHO-5: WHO-5 well-being scale; Depression was assessed using Rimon's Brief Depression Scale (cut-off ≥10); the scale excludes insomnia items and is validated against the Beck Depression Inventory. *P-value is calculated for difference between included and excluded subjects, P<0.05
